# Relationship between marital status and incidence of type 2 diabetes mellitus in a Brazilian rural population: The Baependi Heart Study

**DOI:** 10.1371/journal.pone.0236869

**Published:** 2020-08-03

**Authors:** Camila Maciel de Oliveira, Luciane Viater Tureck, Danilo Alvares, Chunyu Liu, Andrea Roseli Vançan Russo Horimoto, Mercedes Balcells, Rafael de Oliveira Alvim, José Eduardo Krieger, Alexandre Costa Pereira

**Affiliations:** 1 Laboratory of Genetics and Molecular Cardiology, Heart Institute (InCor), University of São Paulo Medical School, São Paulo, Brazil; 2 Department of Integrative Medicine, Federal University of Parana, Curitiba, Brazil; 3 Global CoCreation Lab, Institute for Medical Engineering and Science, Massachusetts Institute of Technology, Cambridge, MA, United States of America; 4 Department of Genetics, Federal University of Parana, Curitiba, Brazil; 5 Department of Statistics, Pontificia Universidad Católica de Chile, Santiago, Chile; 6 Framingham Heart Study, Framingham, MA, United States of America; 7 Department of Biostatistics, Boston University, Boston, MA, United States of America; 8 Bioengineering Department, Institut Quimic de Sarria, Ramon Llull Univ, Barcelona, Spain; 9 Department of Physiological Sciences, Federal University of Amazonas, Manaus, Brazil; Weill Cornell Medical College Qatar, QATAR

## Abstract

Many factors influence the incidence of type 2 diabetes mellitus (T2DM). Here, we investigated the associations between socio-demographic characteristics and familial history with the 5-year incidence of T2DM in a family-based study conducted in Brazil. T2DM was defined as baseline fasting blood glucose ≥ 126 mg/dL or the use of any hypoglycaemic drug. We excluded individuals with T2DM at baseline or if they did not attend two examination cycles. After exclusions, we evaluated a sample of 1,125 participants, part of the Baependi Heart Study (BHS). Mixed-effects logistic regression models were used to assess T2DM incident given different characteristics. At the 5-year follow-up, the incidence of T2DM was 6.7% (7.2% men and 6.3% women). After adjusting for age, sex, and education status, the model that combined marital and occupation status, skin color, and familial history of T2DM provided the best prediction for T2DM incidence. Only marital status was independently associated with T2DM incidence. Individuals that remained married, despite having significantly increased their weight, were significantly less likely to develop diabetes than their divorced counterparts.

## Introduction

Type 2 diabetes mellitus (T2DM) is a multifactorial metabolic disease characterized by the development of insulin resistance and, subsequently, the loss of b-cell function. The worldwide prevalence was 30 million 70 years ago and 108 million 35 years ago [1,2]. It is known that T2DM is rising faster in low-income and middle-income than in high-income countries [1,2]. Brazil has risen from seventh in 1980 to fourth in 2014 in the worldwide country rank of diabetes prevalence (from 2.7 to 11.7 million adults with diabetes) [1].

There are important limitations in generalizing determinants of T2DM incidence from different populations [1–7]. This fact is partially explained by differences in obesity rates, lifestyle, health system resources, and access to medications for preventing the disease [3,8]. The association between marital status and various diseases has been investigated. Especially for T2DM, while some results have highlighted the beneficial effect of marriage [9–11], a poor marital quality may be a unique risk factor in men [12] or being widowed has been associated with a lower risk in women [13]. Moreover, marriage patterns have changed in the last years: people get married later and less often than in the past, there are more divorces and gender roles in a marriage have changed [14], all of which could modify these relationships.

This study aimed to identify the relative importance of socio-demographic variables, in particular marriage status, associated with T2DM incidence in a Brazilian sample from a rural area, after a 5-year follow-up period.

## Materials and methods

### Study population

The Baependi Heart Study (BHS) is a Brazilian cohort that seeks to investigate cardiovascular risk factors and other non-communicable diseases, including both genders aged 18 years old or above. At baseline (cycle 1 from 2005 to 2006), 1,695 individuals in 95 families were recruited in Baependi (19,117 inhabitants, 752 km^2^) located in Minas Gerais State, Brazil [15]. Five years later (cycle 2 from 2010 to 2013), 2,495 individuals from 125 families were evaluated [16]. At each examination cycle, socio-demographic, behaviour, medical history, and physical characteristics were assessed by a standardized protocol. A trained staff collected socioeconomic and clinical data, and all participants were examined in the same research center [15,16].

Of those 2,495 individuals at cycle 2, 1,341 individuals were the same assessed at cycle 1; thus, 354 participants were lost during the follow-up period or died, and 800 were new participants assessed only at cycle 2.

For this study, we carried out the analysis in individuals who attended both examination cycles (n = 1,341). Participants who had some missing data (n = 84, cycle 1; n = 45, cycle 2) were excluded. Individuals with fasting blood glucose ≥ 126 mg/dL or individuals that used hypoglycaemic medications in cycle 1 (n = 87) were also excluded. After exclusions, data on 1,125 diabetes-free individuals in cycle 1 were used to access T2DM incident in cycle 2.

The study protocol was approved by the ethics committee of the Hospital das Clínicas (SDC: 3485/10/074), University of São Paulo, Brazil, and each individual provided informed written consent before participation.

### Sample characteristics

Socio-demographic characteristics included education, marital and occupation status, income, and skin color/race. Those were assessed via interviews using a standardized questionnaire. Education status included four categories: 1) illiterate or never attended school despite reading and writing or attended school for 1 to 4 years; 2) attended school for 5 to 8 years (incomplete or completed primary schooling); 3) attended school for 9 to 11 (incomplete or completed secondary schooling); 4) attended school for more than 11 years or finished university. For analysis, we grouped education into low (categories 1 and 2) or high (categories 3 and 4) levels. Marital status was defined as 1) married, 2) single, and 3) divorced/widower. Occupation status was categorized as 1) employed or retired and 2) unemployed. Since income was very homogeneous in this sample (about 80% of the sample were in the same range of 250–500 dollars/month), we only included occupation status in our analysis. Skin color/race was self-reported (white, brown, black, and indigenous) and stratified into white and non-white for the current analysis.

Social behaviour was also assessed. Smoking status was dichotomized into current/former smokers or never smokers. Alcohol consumption was defined as never drinkers *versus* current or former drinkers.

### Clinical and laboratorial characteristics

Body mass index (BMI) was calculated as body weight (kg) divided by height squared (m^2^). BMI was categorised as normal weight (< 25kg/m^2^), overweight (25 kg/m^2^ to 29.9 kg/m^2^) and obesity (≥ 30 kg/m^2^). Waist circumference was measured half-way between the lowest rib and the iliac crest while the subject was at minimal respiration. Blood pressure (BP) was measured using a standard digital sphygmomanometer (OMRON, model HEM-741CINT) on the left-arm after 5 minutes of rest in the sitting position. Systolic blood pressure (SBP) and diastolic blood pressure (DBP) were calculated from the mean value of three readings.

Hypertension status was defined by the presence of SBP ≥ 140mmHg or DBP ≥ 90mmHg or by the use of antihypertensive medications. Dyslipidaemia treatment was defined by the use of lipid-lowering drugs. Diabetes mellitus was defined as fasting blood glucose ≥ 126 mg/dL or use of hypoglycaemic medications.

Blood collection was standardized, and laboratory assays were conducted in the same clinical chemistry laboratory. The fasting status was declared by the participants at the time of blood collection and the duration of 12 hours was requested.

### Statistical analysis

The incidence of T2DM was assessed after a 5-year follow-up of individuals free of the disease at baseline (n = 1,125 participants).

For the descriptive analysis, categorical variables are presented as percentages and only age is summarised as the mean ± standard deviation (SD). The comparisons of categorical covariates were performed by the Chi-square test, and the means (age) were compared by the Student’s t-test.

Mixed-effects logistic regression models were used to assess the incidence of T2DM adjusting for different characteristics and family (as a cluster variable). The choice to use logistic regression models instead of Cox proportional hazards models was based on the fact that our study included only two visits with the same time interval for all participants [17]. All analyses were corrected for age and sex. Exploratory analyses—sensitivity analyses and a model for diabetes incidence adjusted for BMI change, were conducted post-hoc after identification of marital status as the main socio-demographic predictor for diabetes incidence, in order to search for changes in marital status during a 5-year follow-up and the interaction between sex and BMI changes. All analyses were performed using R version 3.4.2.

## Results

### General characteristics in the Baependi Heart Study

From Table 1, we can see that 57% of participants were women. Approximately 76% of all individuals reported themselves as white and 30% had a familial history of T2DM. More men than women were single (31% vs 25%), smoker (20% vs 12%) and had an occupation with own income (87% vs 57%). At baseline, the mean age was similar for both sexes (41 ± 15 years for women and 43 ± 17 years for men).

**Table 1:** Socio-demographic and clinical characteristics at baseline and 5-year follow-up in the Baependi Heart Study cohort.

Variables	Women 57% (636)		Men 43% (489)	
	Baseline	5-years follow-up	Baseline	5-years follow-up
Age, years	41 ± 15	46 ± 14	43 ± 17	48 ± 16
Race (white, %)	78 (482)	-	75 (358)	-
Familial history (%)	32 (175)	-	28 (104)	-
Education status (low, %)	64 (407)	-	69 (337)	-
Employee/retired (%)	57 (349)	-	87 (423)	-
Marital status (single, %)	25 (158)	16	31 (152)	21
Current/former smoker (%)	12 (79)	-	20 (99)	-
Current/former drinker (%)	32 (205)	-	57 (278)	-
Hypertensive treatment (%)	22 (141)	30 *	15 ^†^ (72)	28 **
Dyslipidaemia treatment (%)	4 (23)	9**	2 (12)	8**
Overweight or obesity (%)	45 (279)	54 *	27 ^†^ (129)	46 ** ^‡^
Altered waist circumference (%)	47 (292)	63 **	17 ^†^ (43)	18 ** ^‡^

Numbers in parentheses refer to the absolute number in each class. Comparisons between baseline and 5-year follow-up

*p-value < 0.05 and

**p-value < 0.001; comparisons between women and men at baseline (†*p*-value) and 5-year follow up (‡ *p*-value). Percentages that did not change over 5-years follow-up are represented by “-”.

Obesity and altered waist circumference increased over time in both sexes. Dyslipidaemia medication use increased approximately 3-fold in both sexes, and hypertension medication use increased by almost 100% in men (15% to 28%).

### Type 2 diabetes mellitus status according to socio-demographic characteristics

The incidence of T2DM was 6.7% in the general sample (75 T2DM in 1125 participants) over 5 years, and there was no significant difference based on sex (7.2% for men and 6.3% for women) (p = 0.63). Based on age groups, the T2DM incidence was 6% (< 29 years); 5.1% (30 to 39 years); 6.4% (40 to 49 years); 6.8% (50 to 59 years); 14% (60 to 69 years); 7% (> 70 years). In the BHS sample, the rate of undiagnosed cases was 30%.

The incidence of T2DM was also analysed according to socio-demographic variables (Table 2). T2DM was more frequent in individuals with high education status, divorced or widower.

**Table 2:** Socio-demographic characteristics according to T2DM status after the 5-year follow-up in the Baependi Heart Study cohort.

Socio-demographic variables	Classes	Non-diabetics	Diabetics at 5-years follow up
Race/skin colour	White (%)	93.7 (787)	6.3 (53)
	Non-white (%)	91.9 (238)	8.1 (21)
Familial history	Yes (%)	93.5 (261)	6.5 (18)
	No (%)	94.2 (613)	5.8 (38)
Education status**	Low (%)	92.9 (691)	7.1 (53)
	High (%)	86.3 (358)	13.7 (22)
Occupation status	Employed or retired (%)	93.7 (723)	6.3 (49)
	Unemployed (%)	92.5 (307)	7.5 (25)
Marital status*	Married (%)	93.8 (662)	6.2 (44)
	Single (%)	94.2 (292)	5.8 (18)
	Divorced or widover (%)	87.1 (88)	12.9 (12)
Smoking status	Current/former smokers (%)	92.7 (165)	7.3 (13)
	Never smokers (%)	93.7 (883)	6.4 (60)
Alcohol consumption	Current/former drinkers (%)	94 (454)	6 (29)
	Never drinkers (%)	92.9 (592)	7.1 (45)

Numbers in parentheses refer to the absolute number in each class. Comparisons of frequencies between non-diabetics and diabetics at 5-years follow up

*p-value < 0.05 and

**p-value < 0.001.

The only socio-demographic variable independently associated with increased odds of presenting diabetes was marital status (Table 3). In our sample, 13% of divorced, 6% of married and 6% of single individuals developed T2DM. After adjusting these estimates for age and sex, being married was associated with a 0.39 odds of developing diabetes; being single was associated with an odds of 0.33 of developing diabetes.

**Table 3:** Age and sex adjusted models for T2DM incident.

	Beta	SE	P value
Skin color (White)	-0.55	0.3	0.08
Occupation	0.14	0.32	0.67
Smoking status	-0.06	0.43	0.89
Alcohol consumption	-0.33	0.30	0.31
Schooling (High)	-0.30	0.36	0.41
Married x divorced	-0.93	0.39	0.02
Single x divorced	-1.10	0.54	0.04

Models corrected by age, sex and follow-up time.

Further investigating this relationship, at baseline, there was no difference in the glucose levels between these three marital status groups, as well as between married and divorced/widower regarding BMI (p value = 0.86), nor between single and divorced/widower (p value = 0.12). In addition, adding baseline BMI to a model predicting diabetes incidence did not significantly change the estimated effect size of marital status suggesting that the observed association is not being mediated by baseline BMI (for being married odds changed from 0.39 to 0.38; for being single odds remained in 0.33).

Nonetheless, the observed association could be mediated by changes in the marital status between baseline and 5-years follow-up. Comparing baseline and 5-years marital status, 63% of individuals remained in their baseline marital status. From the 37% that changed their marital status, the majority of changes occurred in single individuals that have married in the last 5 years (38% of those who changed marital status).

A sensitivity analysis using only individuals that remained in their baseline marital status showed that the estimated effect sizes for being married and being single did not change.

**Fig 1:**
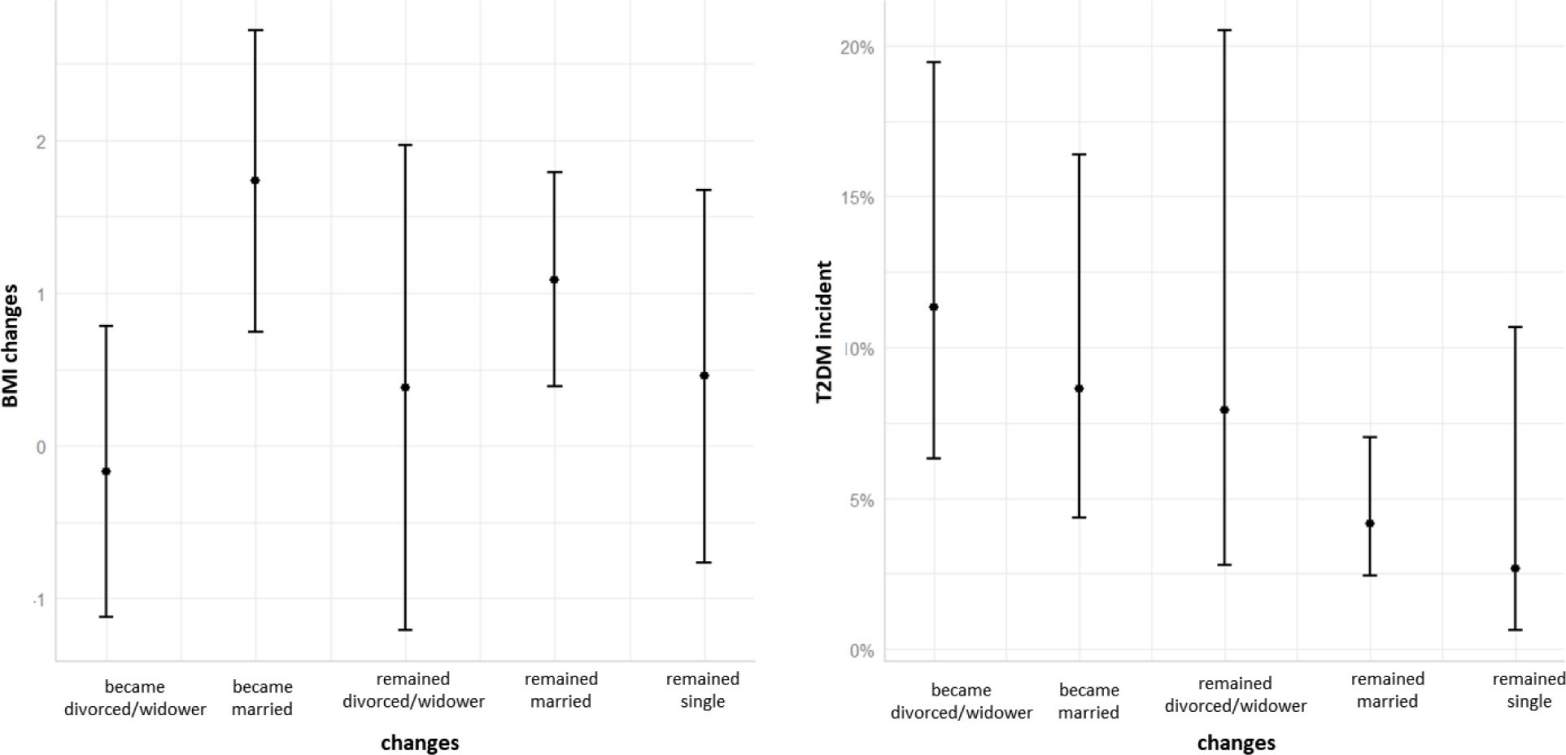
Predicted values of BMI changes and predicted probabilities of T2DM incidence. BMI: Body Mass Index; T2DM: Diabetes Mellitus type 2.

Another analysis, based on a model for diabetes incidence, adjusted for BMI change in addition to all previous potential confounders, showed that the BMI change was highly associated with increased odds of developing diabetes (p value = 0.01), however, its addition did not change the estimated effect size of baseline marital status (OR of 0.39 for being married and 0.31 for being single). Only those that married (p value = 0.0001) or remained married (p value = 0.01) presented significant changes towards increased BMI after 5 years. Despite the increased weight gain, individuals from these groups were still significantly less likely to develop diabetes than divorced/widower individuals (Fig 1).

## Discussion

This is one of the first studies describing T2DM incidence in a sample from a rural city in Brazil. The associations between socioeconomic factors and T2DM occurrence were investigated, and it was possible to identify, among sociodemographic variables, the independent effect of marital status on T2DM incidence.

Baependi is a small city, whose economy is based on family farming. The role of women is predominantly linked to family care. The general characteristic of this sample reflects a typical rural Brazilian population: men had a higher frequency of being single, smoking and a higher rate of employment. It is known that smoking rates are similar between women and men in high-income countries, but the sex difference increases as the country’s income becomes lower [18], as demonstrated in our sample.

We observed a higher prevalence of overweight/obesity among women at baseline, followed by a marked increase in men over the next five years. This seems to have an important relationship with the lifestyle of the population, where in general, women execute the more sedentary activities compared to men, leading to the early onset of overweight/obesity in women. In contrast, men showed a marked decline in their metabolic health later, possibly when they approached the age to retire from rural work. Although this observation is somewhat predictable, it shows the particularities that should be considered in this kind of study, and it may indicate practices for obesity and hypertension prevention, aimed to specific sex and age ranges, that will be more effective since our results are quite different from others who investigated these relationships in urban populations.

Regarding diabetes incidence, in a cross-sectional study, Iser and collaborators found a 6.3% prevalence of self-reported diabetes (5.9% men vs 6.6% women) for the combined population of capitals of Brazil [19]. However, to the best of our knowledge, incidence data for T2DM is still missing for the Brazilian rural population.

The Framingham Heart Study examined T2DM incidence over 8 years within three distinct periods [20]. The age-adjusted 8-year incidence rate of diabetes was higher among men in the 1970s (3.4% vs 2.6%), 1980s (3.6% vs 3.0%) and 1990s (5.8% vs 3.7%) [16]. In the BHS sample, although, there was no statistical difference, the incidence rate of diabetes was also higher in men (7.2% vs 6.3%).

Previous studies have assessed the association between T2DM and socioeconomical factors in the Brazilian population. In an expressive Brazilian sample, in which the prevalence of self-reported T2DM was 7.5%, after adjustments, diabetes remained associated with age (≥ 40 years), education (< 8 years of study), marital status (non-married), obesity, sedentary lifestyle and comorbidity, such as hypertension and hypercholesterolemia [21]. In a specific Brazilian sample, assessed to verify the low adherence to anti-diabetic treatment, including only diabetic patients aged over 20 years, age, female sex and lower income status were associated to T2DM [22]. Other findings have shown that some Brazilian States with greater poverty and lower levels of education had higher rates of T2DM or hyperglycaemia as well [22]. However, these were all based on prevalent cases and mostly self-reported. Here, we add data on predictors for the T2DM incidence rate.

In addition to well-known risk factors for diabetes, such as diet and physical activity, the socioeconomic and sociodemographic factors have shown great importance in this context. The socioeconomic position–measured by educational levels, occupation or income is frequently inversely associated with diabetes [23,24]. Smoking, especially for people with low socioeconomic status, was also identified as a mediator for diabetes development [25]. Although these factors were investigated in our study, the only socio-demographic factor that seemed to have greater importance in predicting the 5-year T2DM incidence in the Baependi population was marital status.

The relationship between marriage and improved health outcome has been previously suggested [26]. Some studies have shown a lower incidence of diabetes [27] and improved adherence to diabetes treatment [28] in partnered patients, since marital relationship influences health behaviours and socioeconomic status. Despite there being no difference in the incidence of T2DM between men and women in our study, the influence of marital status on T2DM seems to be modulated by gender. In a recent study which investigated the diabetes mortality in a large Spanish sample, the highest mortality was observed in divorced/widower women, while single men showed highest mortality [29]. Considering the T2DM incidence, another study found that widowed women compared to married women showed lower risk of T2DM development [13]. In our study, the influence of marital status seemed to be independent of sex.

Our results suggest that, only those who remained married or married during the 5-years follow-up have had a significant weight gain, which was associated with an increased risk of developing T2DM. However, the risk associated to marital status did not change, even after this adjustment. In fact, individuals that remained married, despite having significantly increased their weight, were significantly less likely to develop diabetes than their divorced counterparts.

There are two primary theories that can explain the beneficial effect of marriage on health. The first one is regarding the “selection”: healthier individuals tend to get married and remain married. The second hypothesis corresponds to post-marriage effect: reduction of stress, adoption of healthy behaviours [30–33]. In our study, it is not possible to verify which hypothesis was more coherent, however, probably, both have had an effect on DMT2 development.

In this context, Cornelis and collaborators conducted an important study with a large number of men for ≤22 years and, after various models of adjustment, including lifestyle, BMI, family history, and other variables, widowhood was associated with an increased risk for T2DM in a robust way [34]. In this study, widower and divorced/separated were analysed separately, which is important, as the widowhood and divorce can have different stressful effects [35]. It was reported that the alcohol consumption increased between men who became widower, while both widower and divorced/separated men showed decreased in their BMI and vegetables consumption [35]. Since these factors have influence on T2DM, more studies are necessary to clarify the possible differences regarding the relationship between marital status and T2DM risk.

Some limitations were important in our study context. The lack of adjustment for physical activity as the potential/residual confounding factor is one of them. Additionally, classification of occupation can be mentioned, which may not have been effective in distinguishing participants (lack of distinction for domestic and part-time employees, for example), as well as the marital status, since it was not possible to distinguish widower and divorced/separated individuals.

## Conclusions

In summary, lifestyle influences sex-specific metabolic changes over time. Marital status appears to be a predictor of T2DM incidence and the underlying factors for this association should be further characterised for they may provide important information in the better design and implementation of preventive programs.
